# Cash‐for‐Care Use and Union Dissolution in Finland

**DOI:** 10.1111/jomf.12738

**Published:** 2020-11-17

**Authors:** Kathrin Morosow, Marika Jalovaara, Juho Härkönen

**Affiliations:** ^1^ University of Bath UK; ^2^ University of Turku Finland; ^3^ European University Institute, Stockholm University^,^

**Keywords:** child care, divorce, family policies, longitudinal research, separation

## Abstract

**Objective:**

This study examines how the receipt of the cash‐for‐care (CFC) benefit affects short‐ and long‐term risks of union dissolution.

**Background:**

Several theories predict that couples' gendered division of labor decreases their risk of separation, either due to increased partnership satisfaction or because it establishes economic dependency. Family policies such as the Finnish CFC benefit, which is paid if a young child does not attend public daycare, may encourage such a gendered division of labor, at least temporarily.

**Method:**

Using Finnish register data, this study analyzes the first childbearing unions of 38,093 couples between 1987 and 2009. Discrete‐time event history analyses and fixed effects models for nonrepeated events are applied.

**Results:**

The results suggest a lower separation risk while the benefit is received as compared to couples who do not use it, but no effect in the long‐term. Fixed effects models that control for selection into CFC indicate postponement of separation until after take‐up. Higher‐income mothers show a stronger postponement effect, possibly due to greater income following leave.

**Conclusion:**

CFC use, which signals a temporary gendered division of labor and losses in mothers' earnings, predicts a lower separation risk during receipt of the benefit, but not beyond.

**Implications:**

Policies that affect the division of paid and unpaid labor at best only temporarily reduce dissolution risks.

## Introduction

Family policies have various aims, such as the promotion of maternal employment, enhancing the compatibility of family life and paid work, gender equality, child development, and poverty reduction (see e.g., Mätzke & Ostner, 2010; Thévenon & Gauthier, 2011). In Europe, family stability is rarely a stated family policy goal. Nevertheless, there are theoretical reasons to expect that policies affecting couples' divisions of paid and unpaid labor could either increase or decrease family stability.

Our broad contribution is to analyze whether the use of the cash‐for‐care (CFC) benefit in Finland influences the risk of separation. CFC is paid to parents of children under the age of three who are not in publicly provided childcare, and is primarily used by mothers to care for their children full time. CFC therefore decreases the labor supply of mothers of young children (Hardoy & Schøne, 2010; Kosonen, 2014), and promotes a gendered division of labor between partners, at least temporarily. The associated effect of current CFC use on the risk of union dissolution, however, is theoretically ambiguous. On the one hand, CFC supports a gendered division of couples' paid and unpaid work, a specialization that Becker et al. (1977) argued enhances union stability. Similarly, mothers' drop in income while on CFC increases their economic dependence and so decreases risk of separation (Levinger, 1976; Sayer & Bianchi, 2000). On the other hand, the couple's drop in income while the mother is on leave may induce economic hardship and thus increase separation risks (Oppenheimer, 1997; Sayer & Bianchi, 2000).

Unknown is whether the temporary gendered division of work or reductions in household income while receiving CFC have longer‐lasting effects on union dissolution. Use of CFC may simply postpone the dissolution risk to when the mother returns to work, or it could increase the risk if the mother's absence from employment leads to greater economic stress long‐term. The only study to date found that the introduction of the CFC policy in Norway lowered divorce rates during the first 3 years after childbirth, but did not test effects beyond receipt (Hardoy & Schøne, 2008). Consequently, our second contribution is to differentiate between relative dissolution risk during and after CFC use.

The third theoretical contribution of the paper is to compare effects by mother's income. In most countries, union dissolution risks are greater for less advantaged women (Härkönen & Dronkers, 2006). Differentiating effects of CFC use by women's income levels prior to first birth determines whether CFC use reduces or increases the gradient in dissolution risk among women.

To compare the short‐ and longer‐term impact of CFC receipt on dissolution risk among Finnish mothers, we analyze high‐quality register data from 1987 to 2009 for 38,093 first childbearing cohabiting and married unions of Finnish‐born women. These data allow us to advance empirically on most event history analyses (EHA), by comparing EHA estimates with those from fixed effects models for nonrepeated events (Allison, 2009). Fixed effects models allow us to analyze the timing of union dissolution net of any constant unobserved characteristics that may influence both mothers' CFC use and their risk of union dissolution. By assessing short‐ and long‐term consequences of a family policy reinforcing a gendered division of paid work, the study contributes to the theoretical understanding of partnered women's employment and family stability.

## Cash‐for‐Care in Finland

The Finnish CFC benefit promotes a temporary gendered division of labor in a country where maternal employment and the dual‐breadwinner family are normative. Generally, Finnish men's and women's labor force participation rates are the same, and employment rates are even slightly higher for women (Statistics Finland, 2019a). In addition, both sexes tend to work full time; part‐time work is rare and marginal (Statistics Finland, 2019b). The exception to this gender similarity in employment is that many mothers of young children take long family leaves. Not only do Finnish women have a right to earnings‐related maternity and parental leave, Finland has also offered a further child care benefit (CFC) since 1985.

CFC was a controversial policy when introduced, with opponents arguing it contradicts other policy aims to promote gender equality and maternal employment (Duvander & Ellingsæter, 2016; Hiilamo & Kangas, 2009). Supporters of the policy argued that it gives parents (state‐supported) freedom to choose between family‐based and institution‐based care, extends state childcare support to families (often living in rural areas) that do not benefit from the provision of public daycare, reduces the public costs for childcare, and is in the child's best interest (Hiilamo & Kangas, 2009). As a political bargain, CFC was introduced alongside a subjective right to public daycare for all children under the age of three (Hiilamo & Kangas, 2009).

CFC is paid to parents of children aged 9–35 months who are not in public childcare. Families rarely use CFC to pay a private caregiver or a private daycare provider, as that can be subsidized by another, more generous, allowance (Kosonen, 2014). Instead, one of the parents is the carer in 98% of the families that receive CFC (Salmi et al., 2018). Any family with a child under three is eligible. The CFC system is gender neutral and any leaves can be split between parents; however only one parent can be on leave at any given time. At the time of implementation, CFC could be received simultaneously with unemployment benefits (Anders, 2002), but this possibility was abolished during the economic crisis of the 1990s (Sipilä & Korpinen, 1998). Today, CFC can be combined with other social benefits, but the amounts received are generally affected by these allowances and benefits (Kela, 2018). Despite the length of the leave, employed parents have the right to return to their job after child care leave.

The benefit comprises a basic payment, a means‐tested supplement, possible sibling additions, and municipality top‐ups (Salmi et al., 2017; Sipilä & Korpinen, 1998). The means‐tested supplement is calculated solely on the income of the recipient. In 2017, the average monthly payment per child was €288, and €415 per month per family (Kela, 2017). This contrasts with Finnish women's median monthly earnings of €2,748 in 2016 (Statistics Finland, 2018). Nevertheless, CFC is widely used. In 2016, 87% of eligible families used at least some CFC, and in 97% of these families, it is the mother who is the main carer (Salmi et al., 2018). CFC therefore promotes a temporary gendered division of labor in a country where dual‐earning is normative. There is, however, large variation in how long CFC is used per child. CFC use periods can be extended for additional births during the 3 years, which is not uncommon as CFC use tends to shorten birth intervals (Erlandsson, 2017). On average, mothers with lower socioeconomic status use CFC longer than mothers with higher socioeconomic status (Salmi et al., 2018).

Research on the effects of CFC programs in Finland and other Nordic countries has reported negative effects on mothers' employment and income (Hardoy & Schøne, 2010; Kosonen, 2014; Naz, 2004; Rønsen, 2009; Schøne, 2004). These results suggest that CFC use strengthens women's reliance on their partners' incomes. The research also supports the notion that CFC and other long family leaves reinforce men's breadwinning and mothers' responsibility for care under a gendered division of paid and unpaid work (Duvander & Ellingsæter, 2016; Morgan & Zippel, 2003; Pettit & Hook, 2005; Rønsen, 2001). Parenthood itself results in a more gendered division of domestic tasks (Dribe & Stanfors, 2009; Evertsson & Boye, 2016), which can persist beyond the early parenthood years (Baxter et al., 2008). As a result, policies such as long family leaves that promote a gendered division of labor further reinforce such patterns, which may be sustained beyond the years of take‐up (Chesley & Flood, 2017; Schober & Zoch, 2019). There are competing theories of how a gendered division of household labor affects union stability. By extension, CFC use could have competing short‐ and long‐term effects on dissolution risk.

## Union Dissolution During and After Cash‐for‐Care

CFC is paid to parents of young children. This is a stage in the life course when separation risks are generally low, as having children is indicative of partnership satisfaction and commitment to a shared family life and introduces barriers to union dissolution (Lyngstad & Jalovaara, 2010). Nevertheless, CFC use reinforces a gendered division of labor around the time of the child's birth, a division that can be argued to both decrease and increase dissolution risk. Next, we develop competing expectations from specialization and economic stress theories of how CFC affects union dissolution risks during its use, and why the risks might differ by income. Then, we discuss whether these effects extend beyond the use period, and again vary by income.

### *Union Instability During CFC Use*

Prominent theories of marital stability have emphasized the importance of the household division of labor. According to Becker et al.'s (1977) economic theory of the family, a specialized division of household tasks increases the value of marriage and fosters marital stability. The specialization of one partner (usually the husband) in paid work and the other (the wife) in unpaid household tasks maximizes household efficiency and mutual interdependence (Becker, 1981). Dual‐earning reduces the gains from specialization, increases women's economic independence, and, consequently, increases the risk of divorce (Becker et al., 1977). Later theorists counter that the additional risk of divorce associated with wives' employment can be off‐set if husbands increase their share of domestic tasks (Blossfeld & Müller, 2002; Breen & Cooke, 2005; Cooke, 2006), but we do not have data on couples' unpaid work. In any event, as CFC enforces a gendered division of labor, specialization theory leads us to expect that CFC use reduces the risk of separation of all couples who use it.

Theories on the impact of economic resources on dissolution risk lead to competing hypotheses, as well as possibly varying effects by income. As noted above, most adult Finnish women and men are employed before birth at earnings that greatly exceed CFC benefits. Consequently, using CFC means a considerable drop in household income. This can induce economic hardship, which according to the family stress model can increase conflict between partners and lead to union dissolution as a consequence (Conger et al., 2010). Similarly, Oppenheimer (1997) argued that women's employment improves families' income security and thus reduces the likelihood of union dissolution (also, Cooke, 2006; Ono, 1998; Sayer & Bianchi, 2000). From these perspectives on economic hardship, we expect that use of CFC increases the risk of dissolution.

The potential for economic stress, however, is not shared equally among families. More disadvantaged couples already have fewer resources, so they may experience greater marital strain if there is a drop in income during CFC use. Greater economic hardship is one explanation for the well‐documented educational gradient in divorce risk (Boertien & Härkönen, 2018). The economic hardship effect, thus, is likely to be socially stratified, being more acute among those with weaker economic resources to begin with (Oppenheimer, 1997). Because of this, we would further expect under the family stress model that CFC use increases the risk of union dissolution more among lower‐earning mothers.

Bargaining and exchange models offer competing hypotheses relating to mothers' loss of income. Bargaining theories hold that partners can use their resources, mainly economic ones, to advance their interests within the partnership (Bittman et al., 2003; Breen & Cooke, 2005; Lundberg & Pollak, 1996). Economic resources also facilitate exiting the partnership if the bargaining does not lead to a satisfactory outcome (Bittman et al., 2003; Breen & Cooke, 2005). CFC use typically reduces the female partner's economic resources and increases her economic dependence on the male partner. Mothers' greater economic dependence can create barriers to ending a partnership by increasing the perceived costs of a separation (Levinger, 1976; Sayer et al., 2011; Sayer & Bianchi, 2000; Schoen et al., 2002). Economic dependence under bargaining models, therefore, suggests that CFC use reduces the likelihood of partnership dissolution. Less clear is whether dependence effects vary with women's income. Lower‐income women have fewer resources, but CFC payments reflect a larger proportion of their foregone and potential earnings than for high‐income women. Arguably, then, (at least temporary) dependence effects are larger for higher‐income women, who have steeper loss in income during CFC use.

Hitherto, only one empirical study has analyzed the short‐term effects of CFC use on divorce (Hardoy & Schøne, 2008). The authors of this Norwegian study found the introduction of CFC reduced dissolution risk by one percentage point, a rather considerable effect given that approximately 4% of married couples divorced during the 3‐year follow‐up (Hardoy & Schøne, 2008). They did not assess whether effects varied among women. Neither did they consider longer‐term consequences of CFC use.

### *Union Instability After CFC Use*

Couples' divisions of paid and unpaid work, as well as dissolution risk, vary over the life course. CFC use is restricted to a specific period in life, but may affect the timing of separation or risk of separation in the long run. (For related arguments on women's employment and divorce, see Özcan & Breen, 2012; Killewald, 2016).

Given the children's young age and that the low level of benefit increases women's economic dependence, a partner with an intention to separate may decide to postpone the separation until after the CFC use, when the majority of mothers enter or return to employment. Such postponement is indicated if the union dissolution risk is lower during CFC use, but the risk increases above the baseline level afterwards.

Postponement likely varies with women's income. Less advantaged women endure greater marital strain due to economic hardship (Boertien & Härkönen, 2018). Nevertheless, their overall economic insecurity unlikely increases considerably with CFC use because women with poor labor market prospects are more likely to be economically dependent (e.g., on welfare or family members) whether they are partnered or not (Oppenheimer, 1997). For higher earning women, conversely, CFC use represents a much greater drop in income while on leave. Hence, although low‐earning women are more likely to have a greater dissolution risk in the short and long‐term, we expect higher‐earning women to have a stronger incentive to postpone the separation beyond CFC use because their independent economic resources will increase more after the leave. In other words, the increase in divorce risk after CFC use will be greater for high‐ than low‐earning women.

Extended family leaves also lead to a loss in mothers' work experience, which can have negative longer‐term consequences on their labor market position (Rønsen, 2009). A weaker position in the labor market can increase union dissolution risk in the long‐term. If a weaker position in the labor market of mothers leads to economic stress and conflict between partners, union dissolution risks may increase after CFC use. As above, the risk should then be greater for lower‐income women as they already face greater economic stress.

The theoretical expectations are summarized in Table 1.

**Table 1:** Summary of Theoretical Expectations for the Effect of CFC on the Risk of Union Dissolution

		Expectations for union dissolution risk	Income differences (mother)
During CFC	Specialization	Decrease	No difference
	Economic stress	Increase	Low‐income: greater risk
	Economic dependency	Decrease	High‐income: greater risk
After CFC	End of economic dependency	Increase (postponement)	High‐income: greater risk
	Long‐term economic stress	Increase	Low‐income: greater risk

## Method

### *Data and Variables*

We used register data from an 11% random sample of persons born between 1940 and 1995 who had been recorded in the population of Finland between 1970 and 2009. The data were compiled by Statistics Finland by linking information from various administrative and longitudinal population registers. They include full monthly histories of co‐residential partnerships regardless of marital status (from 1987 onwards; for rules of inference of cohabitations, see Jalovaara and Kulu (2018)), monthly histories of childbearing and other vital events, education, and yearly employment and income data from various sources.

Our analytical sample consists of unions of Finnish‐born women in which they had their first biological child. The data covers the years from 1987 to 2009, and the couples entered the data between 1988 and 2007, that is, during the years in which we could observe their first births and their CFC use. On average we follow these couples for 6.3 years, all of the couples were eligible to receive CFC. The analyses included 38,093 couples of which 9,014 separated, contributing 312,256 couple‐years at risk.

The dependent variable is the dissolution of the woman's first childbearing union in a given year—defined as partners permanently moving apart or divorcing, whichever came first. Because families become eligible for CFC once the child is 9 months old, and because we lag the CFC use variable by 1 year (see below), the couples become at risk of union dissolution on the second calendar year after the first child is born. The unions are right‐censored if the woman emigrates, either partner dies, or at the end of the observation period (September 2009).

Our main independent variable is a time‐varying measure of CFC use and time since the latest use. The variable is lagged (by 1 year) to avoid confusing the time‐ordering of CFC receipt and separation. This variable is based on annual data of the received amount; we do not have information on the exact duration of the CFC use in a given year. Therefore, for the main analysis, the time‐varying CFC use variable includes the following categories: no previous CFC use (reference category), current CFC use, 1 year since, 2–4 years since, 5–7 years since, and 8+ years since the latest CFC receipt. Categories indicating years since the latest use were included to estimate whether CFC use affects the union dissolution risk beyond the take‐up.

We included three groups of control variables. The first comprises duration (linear and squared), period (calendar year) in groups based on economic cycles, and union duration at the birth of first child (in years). The second group includes variables measuring the couple's sociodemographic profile: age (of the female partner) in 10‐year age groups (time‐varying); education of both partners (time‐varying), categorized as basic, secondary vocational, secondary academic, lower tertiary, and higher tertiary; union type prior to first birth (marriage or cohabitation); region of residence (time‐varying), differentiating urban, semiurban and rural municipalities; the number of months the mother had been employed the year prior to the first birth; partners' unemployment (time‐varying dummy), and mother's income prior to first birth deflated to the euro value in 2011, collapsed in the following categories: less than 10,000, 10,000–15,999, 16,000–27,999, more than 28,000. Third, because CFC receipt is tied to the age of the youngest child and the length of the take‐up is related to the number of children, both were included as additional control variables. The age of the youngest child (time‐varying) was measured as: less than 1 year (when most mothers are on maternal and parental leave), between 1–2 years (when the couple is eligible for CFC), and 3+ years (not eligible), and the number of children (time‐varying) was measured as 1, 2, 3, and 4 or more children. All time‐varying control variables were lagged by 1 year. Table 2 shows the distributions of these variables over the 312,256 couple‐years at risk of the 38,093 couples in the sample.

**Table 2:** Total Exposure Time (*N* and %) Spent in Different Categories of the Background Variables; Finnish Women's First Childbearing Unions Between 1987 and 2009

		Couple‐years at risk	
		In 100s	Percent
CFC use and time since CFC use (tv)	No CFC	314	10
	Currently using CFC	1,213	39
	1 year since CFC	343	11
	2–4 years since CFC	621	20
	5–7 years since CFC	334	11
	8+ years since CFC	297	9
Length of CFC use (tv)	0 years	314	10
	1 year	672	22
	2 years	597	19
	3 years	538	17
	4 years	357	11
	5 years	271	9
	6+ years	374	12
Period (tv)	1987–1990	25	1
	1991–1994	152	5
	1995–1997	312	10
	1998–2000	648	21
	2000–2004	885	28
	2005–2009	1,101	35
Age, female partner (tv)	Under 21	12	0
	21–30	967	31
	31–40	1,682	54
	41–50	437	14
	51+	24	1
Female partner's education (tv)	Basic	277	9
	Secondary vocational	1,121	36
	Secondary academic	204	6
	Low tertiary	1,076	35
	High tertiary/university	445	14
Male partner's education (tv)	Basic	501	16
	Secondary vocational	1,328	43
	Secondary academic	164	5
	Low tertiary	722	23
	High tertiary/university	407	13
Union type prior to 1st birth	Cohabiting	1,548	50
	Married	1,574	50
Region of residence (tv)	Urban	2,104	67
	Semiurban	526	17
	Rural	492	16
Months employed prior to 1st birth, female partner	0 months	607	19
	1–5 months	140	5
	6–11 months	179	6
	12 months	2,195	70
Male partner unemployed (tv)	No	2,908	93
	Yes	215	7
Income prior to 1st birth, female partner	<10,000	706	23
	10,000–15,999	640	20
	16,000–27,999	1,373	44
	≥ 28,000	402	13
Number of children (tv)	1	998	32
	2	1,497	48
	3	491	16
	4+	137	4
Age of the youngest child (tv)	0	385	12
	1–2	1,093	35
	3+	1,644	53
		Mean	SD
Union duration in years prior to 1st birth		2.94	2.3
Outcome event: Separation	No	3,032	97
	Yes	90	3
Couple‐years at risk		312,256	
Couples (*N*)		38,093	

*Source:* Finnish Register Data, own calculations.

*Note:* All time‐varying (tv) independent variables are lagged by 1 year.

### *Analytical Strategy*

Our analysis followed three stages. First, we fit logistic regression models to estimate the effects of CFC use on the union dissolution risk using yearly discrete‐time event history data. Mothers are followed from when they were first eligible to receive CFC. We estimated three models: The first controlled for duration since first eligible for CFC, period, and duration of the union prior to the first birth. The second added controls of the couple's sociodemographic characteristics. The third model added the age of the youngest child and the number of children. Given how our CFC use variable is constructed, the analysis describes whether the union dissolution risk differs during and after CFC, as compared with mothers who had not used CFC by that year.

Despite our rather extensive list of control variables, the estimates of the effects of CFC on union dissolution can be confounded by unobserved factors, such as unobserved human capital, values or family orientation, that affect both CFC use and the risk of union dissolution. Thus, second, we used fixed effects discrete‐time event history models for nonrepeated events (Allison, 2009; Allison & Christakis, 2006) that control for time‐constant unobserved factors. Following the strategy used by Allison and Christakis (2006), we used the fixed effects models to measure union dissolution risk during CFC use and in subsequent years by estimating separate models for each subepisode (i.e., during CFC use, 1 year after, 2–4, 5–7, and 8–10 years after). These models estimated the union dissolution risk at each episode compared to all other episodes (cf. Allison & Christakis, 2006). The fixed effects models included the same time‐varying controls as the regular models to adjust the estimates of CFC on separation (Allison, 2009).

Fixed effects models control for unobserved heterogeneity, but with the cost that they cannot be estimated with the same sample as the ordinary event history models. Specifically, the former are estimated using conditional logit models from cases with over‐time variation in the independent variable. In our case, couples who did not use CFC during the follow‐up period (9%), or used it during all the observed years (20%) were excluded from this analysis. Couples thus vary only in how many years—not whether—they used CFC. To better compare the results between fixed effects and ordinary event history models—in order to assess bias from unobserved heterogeneity—we estimated models on the same sample as in the fixed effects analysis using ordinary event history models with logistic regression, with the same control variables as above. We compared the results statistically using the Hausman test; as an additional check, re‐ran the analysis by estimating a random effects model on the same sample and reached the same conclusions. Fixed effects models controlled for confounding from time‐constant factors, but as is the case with all fixed effects models, the method does not control for time‐varying unobserved variables or the effects of anticipation of separation on labor supply (cf. Oppenheimer, 1997; Özcan & Breen, 2012).

In the final step of the analysis, we included interaction effects between CFC use and the mother's income prior to first birth to assess whether any effects are moderated by socioeconomic position as well as to help disentangle the mechanisms behind a possible effect. We used mother's income prior to first birth as our main measure as it is the most proximate measure of her (expected) income and economic independence, but replicated the findings using her education likewise. These interaction effects were included in both the regular and the fixed effects event history models.

## Results

Nine percent of mothers in our sample did not receive CFC following the birth of any of their children. This is consistent with previous findings that a large majority of Finnish mothers use the benefit for at least some time after the parental leave period. In addition to mothers that never received CFC during the follow‐up period, the reference category in the time‐varying “CFC use” variable (see Table 3) includes the years when a couple has not yet used any CFC. The composition of this category of mothers was similar to other mothers except that they are, on average, slightly higher educated, have slightly higher income and fewer children.

**Table 3:** Cash‐for‐Care (CFC) Use and Separation Risk, Odds Ratios From Discrete‐Time Event History Models

		Model 1		Model 2		Model 3	
CFC use and time since CFC use (tv)	No CFC	1		1		1	
	Currently using CFC	0.81	***	0.69	***	0.85	***
	1 year ago	1.07		0.97		1.01	
	2–4 years ago	1.16	***	1.06		0.99	
	5–7 years ago	1.29	***	1.20	***	1.03	
	8+ years ago	1.29	***	1.24	**	0.98	
Duration		0.92	***	0.96	***	0.98	
Duration squared		1.00		1.00		1.00	
Period (tv)	1987–1990	0.95		0.83		0.82	
	1991–1993	0.88	*	0.84	**	0.84	***
	1994–1996	0.79	***	0.77	***	0.78	***
	1997–2000	0.80	***	0.78	***	0.78	***
	2001–2004	0.83	***	0.81	***	0.81	***
	2005–2009	1		1		1	
Union duration prior to 1st birth		0.87	***	0.94	***	0.94	***
Age, female partner (tv)	Under 21			1.87	***	1.84	***
	21–30			1		1	
	31–40			0.78	***	0.74	***
	41–50			0.66	***	0.57	***
	51+			0.51	***	0.39	***
Female partner's education (tv)	Basic			1.50	***	1.46	***
	Secondary vocational			1		1	
	Secondary academic			1.10	*	1.09	*
	Low tertiary			0.83	***	0.84	***
	High tertiary/university			0.75	***	0.78	***
Male partner's education (tv)	Basic			1.32	***	1.30	***
	Secondary vocational			1		1	
	Secondary academic			0.99		0.99	
	Low tertiary			0.79	***	0.80	***
	High tertiary/university			0.72	***	0.74	***
Union type prior to first birth	Cohabiting			1.29	***	1.24	***
	Married			1		1	
Region of residence (tv)	Urban			1		1	
	Semiurban			0.76	***	0.77	***
	Rural			0.66	***	0.67	***
Months employed prior to 1st birth, female partner	0 months			1.14	***	1.14	***
	1–5 months			1.04		1.03	
	6–11 months			1.03		1.03	
	12 months			1		1	
Male partner unemployed (tv)	No			1		1	
	Yes			1.48	***	1.47	***
Income prior to 1st birth, female partner	<10,000			1.19	***	1.19	***
	10,000–15,999			1.09	*	1.08	
	16,000–27,999			1		1	
	≥28,000			1.04		1.05	
Age of the youngest child (tv)	0					0.50	***
	1–2					0.77	***
	3+					1	
Number of children (tv)	1					1	
	2					0.79	***
	3					0.67	***
	4+					0.59	***
Constant		0.08	***	0.06	***	0.08	***
Log‐likelihood		−40,383.3		−39,293.9		−39,032.7	
LR chi^2^				2,178.8	***	522.5	***
Observations		312,256		312,256		312,256	

*Source:* Finnish Register Data, own calculations.

*Note:* All time‐varying (tv) independent variables are lagged by 1 year. (tv) indicated time‐varying independent variable.

*p < .05. **p < 0.01. ***p < .001.

### *Discrete‐Time Event History Models*

Table 3 shows the results from the discrete‐time event history models on CFC use and the risk of union dissolution. The time‐varying independent variable distinguished between no (current or previous) use of CFC, current CFC use and time since the latest use. The first model in Table 3 includes controls for duration, period, and the duration of the union prior to the first birth; the second model also controls for the partners' socioeconomic characteristics; and the third model adds controls for the number of children and the age of the youngest child.

The results from the first model indicated that couples currently using CFC have an approximately 20% lower rate of separation than couples with neither current nor previous use of CFC (Table 3, Model 1). The separation risk increases during the years after CFC use. This main result remains when controlling for the partners' sociodemographic characteristics (Model 2). The difference between couples currently using CFC and those who had not used it previously increases (odds ratio = 0.69), suggesting that couples who are more likely to use CFC have characteristics that also increase their separation risk. Lower age and educational attainment are prime candidates of such characteristics (Jalovaara & Kulu, 2018). The estimates for the years after CFC use are smaller in Model 2 than in Model 1. The positive and significant estimates for 5 or more years after CFC use suggest a postponement effect, meaning that although CFC users have lower union dissolution rates while using the benefit, they “catch up” later.

After adding controls for the number of children and the age of the youngest child (Model 3), the estimate for CFC use is reduced and the estimates for the years since CFC use are close to 1 and no longer statistically significant. By construction of the policy, CFC use overlaps with the time when children are young, which is also the time when separation rates are low in any case. Although this confounds part of the CFC use effect, Model 3 shows that couples still have a 15% lower separation risk while they are using CFC compared to couples who have not used it previously. The “catch‐up” effect after CFC use (found in Models 1 and 2) is completely confounded; the years after CFC use overlap with the time when the youngest child is beyond the toddler‐age and separation rates are higher. Overall, the results from Model 3 do not support a postponement effect but point to a small temporary reduction in union dissolution risks during CFC use, which then returns back to the baseline level after the use period.

Additional analyses (shown in the online supplement, Figure S1) assess the longer‐term effects of CFC use on union dissolution by estimating the survival functions for two hypothetical couples with two children born 2 years apart using the reference values of the independent variables, but varying CFC use. Figure S1 shows that because CFC use is generally restricted to a short period of time in a specific life stage, its small suppressing effect on union dissolution has minimal importance on the likelihood of union dissolution over 15 years. An alternative model specification using CFC use, time since CFC use as well as accumulated length are presented in the online supplement as well (Table S1). Table S1 shows that controlling for leave length does not change the effect of CFC use in the long run.

### *Fixed Effects Models*

The estimates from the above analyses can be biased due to selection by unmeasured factors; for instance, values and fertility preferences may affect CFC use and also be correlated with separation risk. We estimate a series of fixed effects discrete‐time event history models for nonrepeated events to control for unobserved factors that do not vary over time. As discussed in the methods section, we estimated separate models for CFC use and the time periods after the latest CFC use. The remaining time points, next to the one of interest, form the reference category for each model. As also discussed in the methods section, the analytical sample for the fixed effects models consists of couples which were observed to use CFC for some, but not all years (which comprises 71% of the couples).

The upper panel in Table 4 presents the results from the fixed effects models (full table in appendix Table A1). CFC use reduces the separation rate by 41% compared to the other periods. The separation risk increases steadily in the years after CFC use, peaking at 5–7 years post use, and later stabilizes to the baseline. This suggests a postponement effect, in which the separation risk is temporarily suppressed during CFC use, only to increase in the years after. In the absence of such an effect, one would expect a stable separation risk during the years after CFC use.

**Table 4:** Event History and Fixed Effects Results for Nonrepeated Events (Odds Ratios) With Varied Measures of the Timing of CFC Use on the Risk of Union Dissolution

		Union dissolution after CFC receipt			
	Union dissolution during CFC receipt	1 year	2–4 years	5–7 years	8–10 years
Fixed effects estimate*s*					
Odds ratio	0.59	1.23	1.34	1.42	1.12
*p* value	.00	.00	.00	.00	.24
Event history analysis estimates					
Odds ratio	1.04	1.16	1.08	1.11	0.92
*p* value	.30	.00	.01	.01	.19
Hausman test (*df*)	6,798.49 (28)	861.36 (28)	3,287.51 (26)	133.25 (24)	1,092.82 (23)
*p* value	.00	.00	.00	.00	.00

*Source:* Finnish Register Data, own calculations.

*Note:* Models controlled for female partner's age and age squared, both partners' education, age of the youngest child, number of children, region of residence, period and male partner's unemployment—all variables lagged by 1 year. Event history models additionally controlled for Union type and duration prior to first birth, income prior to first birth and employed months prior to first birth.

The middle panel shows the estimates from regular discrete‐time event history models on the same data as used to estimate the fixed effects models. These estimates are more directly comparable to the fixed effects ones than the estimates presented in Table 3. According to these results, the separation rate during CFC use does not differ from the other periods and the separation rate increases modestly in the years after CFC use. Note that the coefficient for “during CFC” is not statistically significant with the change in the sample and baseline group (the sample does not include couples who never received CFC). The test statistics of the Hausman test, in the lowest panel, indicate that the differences between the ordinary and fixed effects estimates are statistically significant, implying unobserved heterogeneity. The difference between the estimates suggests that couples which use more CFC have higher separation rates due to unobserved factors than couples which use CFC for shorter durations. Such unobserved factors may include unmeasured differences in human capital (e.g., Salmi et al., 2018), among other reasons, which are associated with longer CFC use spells as well as higher separation rates. This selection of couples with higher separation risks into more CFC use biases the estimates of CFC use. Specifically, it underestimates the postponement effect of CFC, as shown by the suppression of the negative effect of current CFC use and of the positive effect of the years after CFC use.

### *Do the Effects Vary by Income?*

The results presented thus far indicate a temporary effect of CFC take‐up whereby separation risk is reduced while using CFC, but also point toward a postponement of separation until after CFC use. Results also indicate that less advantaged mothers are more likely to use CFC and use it for longer (Salmi et al., 2018), furthermore they are more likely to separate (Härkönen & Dronkers, 2006). It is unclear, however, whether CFC use affects separation risks among high or low earning mothers differently. As discussed above, low earning mothers may show higher separation risks and economic constraints overall that are less affected by CFC use, whereas high earning mothers may show a stronger postponement effect as their expected earnings following CFC use are much higher.

**Figure 1:**
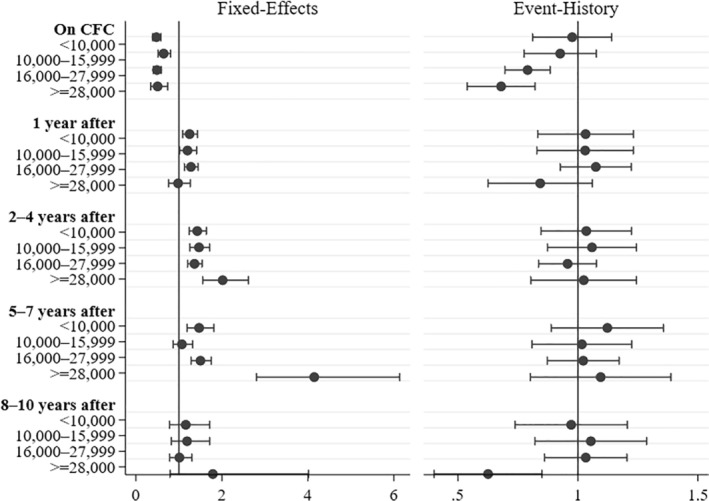
Effects of CFC Use on Union Dissolution by the Mother's Income Prior to First Birth. Discrete‐Time Event History and Fixed Effects Discrete‐Time Event History Models for Nonrepeated Events. *Note:* The Estimates Are Predicted Based on an Interaction Model Between Mother's Income Prior to First Birth and the CFC Use Variable. The Event History Model Controlled for Duration, Duration Squared, Period (tv), Union Duration Prior to 1st Birth, Female Partner's Age (tv), Both Partners' Education (tv), Union Type Prior to First Birth, Region of Residence (tv), Female Partners' Months Employed Prior to 1st Birth, Male Partners Unemployment (tv), Age of the Youngest Child (tv), and the Number of Children (tv). Fixed Effects Event History Models Controlled for Female Partner's Age, and Age Squared (tv), Both Partners' Education (tv), Age of the Youngest Child (tv), Number of Children (tv), Region of Residence (tv), Period (tv) and Male Partner's Unemployment (tv). (tv) Indicated Time‐Varying Independent Variable, Lagged by 1 Year. *Source:* Finnish Register Data, own calculations.

In order to better understand the temporary reduction of separation risk, models including an interaction term between CFC use (including time since use) and the mother's income prior to first birth were estimated for both the event history models and the fixed effects models. The income categories were defined through percentiles, representing a 25, 50 and 90 percent cut off. The highest category of above 28,000 euro is the smallest group, but still spans over 6,000 couples and 13 percent of the couple‐years at risk. Figure 1 shows the predicted point estimates and confidence intervals of the effects of CFC use and the following years, based on linear combinations from the interaction model.

Inclusion of the interaction term between CFC use and income into the regular event histoegry model improves the model fit (LR test = 34.32; *df* = 15; *p* = .0031). The interaction results indicate that the temporary reduction in separation risk while CFC is received is limited to higher earning mothers, and that there are no differences by income in the effect in the years after CFC use (Figure 1, right panel).

Figure 1 further shows the results of the interaction effects for the fixed effects models (left panel). These results indicate that, among only mothers who used CFC, current CFC use lowers separation risks regardless of the female partner's previous income level. This is further reflected in that the inclusion of the interaction term improves the model fit only for the models observing the years 5–7 (LR = 32.30; *df* = 3; *p* = .00) after the latest CFC receipt. One year after CFC use a slight increase in separation risk can be seen for all income groups but the highest. Two to four years after the latest CFC receipt, the separation risk across all income categories increased slightly. This increase is not equal for all income groups; instead we find the starkest effect for the highest income group. Five to seven years after CFC use, the strongest effect can again be seen among higher earning mothers. Postponement of separation until 5–7 years after the leave is, therefore, more marked for couples where her previous income was high.

## Conclusions

The relationship between women's employment and separation has been the focus of many studies. In a gender‐egalitarian context such as Finland, mothers' employment rates are close to those of fathers', and policies that negatively affect maternal employment, such as the CFC benefit, can contradict the more general policy aims of gender equality. The CFC policy indirectly promotes at least a temporary gendered division of labor within couples and leads to longer periods outside the labor market among young mothers that can result in loss of economic and human capital. This study used Finnish register data from 1987–2009 to analyze the effects of CFC use on union dissolution.

We estimated discrete‐time event history models, with and without fixed effects that control for unobserved time‐constant factors. The results from the regular event history models without fixed effects show that, net of the effects of all control variables, separation rates are lower while CFC is received, but there is no effect in the years following the take‐up compared to couples who have not used CFC. This suggests that any effect of CFC use is temporary and does not alter couples' separation rates in the long run. Hardoy and Schøne (2008) estimated that the introduction of CFC reduced the union dissolution risk by one percentage point, or by 25%, in the short term (3 years). Our results suggest that such effect is only temporary and overshadowed by other factors that affect union dissolution in the long term.

These results could, however, be biased due to direct and indirect selection into CFC use. To control for time‐constant unobserved factors, we estimated fixed effects models for nonrepeated events. The estimates from these models generalize to a different population—excluding couples that either never used CFC, or used it during all observed years—and use within‐couple rather than between‐couple variation like the estimates from ordinary discrete‐time event history models. The results from the fixed effects models suggest that CFC use has a stronger negative effect on the separation risk than in the previous discrete‐time models. However, this union stabilizing effect is only temporary, and is followed by a postponement or catch‐up effect in which separation risk increased after its use. A comparison between the fixed‐ and ordinary discrete‐time event history models furthermore suggests a strong selection into (longer episodes of) CFC use, in which couples with a higher unobserved separation propensity use CFC more than those with a lower separation propensity. A likely explanation for our result is unmeasured socioeconomic characteristics: Finnish couples with a weak socioeconomic profile are more likely to use CFC, and are also more likely to subsequently separate (Jalovaara & Kulu, 2018). This interpretation is in agreement with the results from the conventional event history models, in which controlling for observed socioeconomic characteristics increased the effect of CFC use. Although the fixed effects models control for unmeasured stable characteristics, the estimates could remain biased if, for example, decisions on CFC use are driven by anticipation of separation. A further limitation of our analysis is that we measured CFC use based on annual data, not data on exact CFC spells, which can introduce measurement error into the models. However, we do consider this to be much less of a problem for our measure of time since CFC use.

All in all, our results are best interpreted as CFC imposing a temporary reduction in separation risk and that couples may merely postpone separation. Our results of a lower separation risk during CFC use are in line with theories suggesting a lower separation risk when couples specialize in either paid or unpaid work (Becker, 1981). These findings also support the argument that taking‐up CFC could lead to economic dependency of a partner and thus create barriers to leaving a partnership. As CFC is a low‐paid benefit, women might decide to wait until they are back in the labor market before separating.

Theoretical arguments for the long‐term effect of CFC following its use were ambiguous. While we would assume a postponement effect if economic dependence creates barriers to separation, a loss in human capital through CFC use could increase separation risks long term due to economic strains. An interaction effect showed that independent of prebirth earnings all mothers show lower separation risks during CFC use. The postponement effect seems to be somewhat stronger among higher earning mothers, however. These mothers experience a larger drop in earnings during CFC take up, but tend to have a higher earnings potential after the leave, which provides them with financial resources that facilitate leaving an unsatisfactory partnership. Note, however, that job protection for parents who take family leave enables postponement as parents have a job to return to. Contexts that do not provide this protection may find less of an postponement effect. Future research with data containing gender ideology and domestic work measures might assess whether mothers who postpone separation have more egalitarian views and whether a potentially more gendered division of labor following the CFC use might leave them increasingly dissatisfied.

By focusing on the short‐ and long‐term effects of a specific policy on union dissolution, we underline the potential influence that policy has on family stability. A temporary reduction in separation risk while receiving the CFC can be both beneficial and unfavorable. Lower separation rates during these years might be favorable for children, parents and their bonds, given the children's young age. However, if there is a postponement effect due to mothers' lack of economic resources, which prevents the partners from dissolving a high‐conflict union, the well‐being of all family members may be decreased (Sayer & Bianchi, 2000). Thus, even in a country context in which employed women are overall less likely to separate (Cooke et al., 2013), policies that affect the division of paid and unpaid labor have the potential to affect families and union dissolution risks.

## Note

The research leading to these results has received funding from the Strategic Research Council of the Academy of Finland (Decision Number 293103) for the research consortium Tackling Inequality in Time of Austerity, the Academy of Finland (grant 275030, 321264 and 320162), the Swedish Research Council through the Linnaeus Center for Social Policy and Family Dynamics in Europe (grant 349‐2007‐8701), the Swedish Initiative for Research on Microdata in the Social and Medical Sciences (SIMSAM): Stockholm University SIMSAM Node for Demographic Research (grant 340‐2013‐5164) and the European Research Council (ERC) under the European Union's Horizon 2020 research and innovation program (grant agreement No. 680958, L. P. Cooke, PI). We would like to thank Sunnee Billingsley, Ann‐Zofie Duvander, Rense Nieuwenhuis, Peter Fallesen and Lynn Prince Cooke for valuable comments, and Josh Blamire for proofreading.

## Supporting information


**Appendix S1:** Supporting Information

## Appendix

**Table A1:** Event History and Fixed Effects Results for Nonrepeated Events (Odds Ratios) With Varied Measures of the Timing of CFC Use on the Risk of Union Dissolution

		Fixed effects estimate										Event history analysis estimates									
		Union dissolution during CFC receipt		Union dissolution after CFC receipt								Union dissolution during CFC receipt		Union dissolution after CFC receipt							
				1 year		2–4 years		5–7 years		8–10 years				1 year		2–4 years		5–7 years		8–10 years	
Separation risk		0.59	*******	1.23	*******	1.34	***	1.42	***	1.12		1.04		1.16	***	1.08	*	1.11	*	0.92	
Duration		0.24	***	0.78	*******	1.47	***	4.81	***	10.44	***	0.47	***	1.02	**	1.70	***	4.81	***	10.09	***
Duration squared		1.04	***	0.99	*******	0.96	***	0.94	***	0.93	***	1.02	***	0.98	***	0.96	***	0.93	***	0.93	***
Period (tv)	1987–1990	1.35	*	0.60	*******	0.00		0.00		0.00		0.73	***	0.54	***						
	1991–1993	1.27	**	0.92		0.96		0.00		0.00		0.89	***	0.86	***	0.75	***				
	1994–1996	0.86	*	1.06		1.29	***	0.95		0.00		0.73	***	0.98		1.05	*	0.86	**		
	1997–2000	0.87	**	1.01		1.29	***	1.27	***	1.30	**	0.76	***	0.91	***	1.09	***	1.13	***	0.99	
	2001–2004	1.03		0.96		1.05	*	1.13	***	1.10	*	0.90	***	0.88	***	0.94	***	1.13	***	1.03	
	2005–2009	1		1		1		1		1		1		1		1		1		1	
Union duration prior to 1st birth												1.00		1.01	***	1.01	**	1.00		1.00	
Age, female partner (tv)	Under 21	2.04	***	0.17	*******	0.00		0.00		0.00		1.78	***	0.19	***						
	21–30	1		1		1		1		1		1		1		1		1		1	
	31–40	1.15	***	1.03		1.22	***	1.63	***	2.45	***	1.19	***	1.04	*	1.20	***	1.46	***	2.18	***
	41–50	0.80	***	0.82	*******	1.07		1.48	***	2.46	***	1.06	***	0.73	***	0.95	*	1.45	***	2.29	***
	51+	0.11	*	0.04	**	0.14	***	0.25	***	1.01		0.07	***	0.03	***	0.13	***	0.38	***	1.48	**
Female partner's education (tv)	Basic	1.51	***	1.33	**	0.60	***	0.68	**	0.80		1.48	***	1.01		0.91	***	0.90	***	0.83	***
	Secondary academic	1.23	*	1.15		0.59	***	0.54	***	0.50	**	0.97		1.07	**	0.99		0.93	*	0.93	
	Secondary vocational	1		1		1		1		1		1		1		1		1		1	
	Low tertiary	1.15		1.00		0.80	***	0.99		1.20		0.74	***	1.08	***	1.05	***	1.04	*	1.12	***
	High tertiary/university	0.71	**	1.16		0.97		1.12		0.77		0.51	***	1.21	***	1.20	***	1.09	**	1.16	***
Male partner's education (tv)	Basic	1.31	*	0.76	*	0.76	*	1.24		0.90		1.07	***	0.98		0.97		0.98		0.99	
	Secondary academic	1.37	*	0.94		0.82		0.84		0.98		1.01		0.97		0.97		0.97		0.98	
	Secondary vocational	1		1		1		1		1		1		1		1		1		1	
	Low tertiary	1.22	*	1.10		1.07		0.92		0.85		0.95	**	1.02		0.99		0.98		1.03	
	High tertiary/university	1.48	**	0.91		0.91		0.92		0.94		1.01		1.02		0.98		0.97		0.98	
Union type prior to 1st birth	Cohabiting											0.95	***	1.10	***	1.11	***	1.02		0.96	*
	Married											1		1		1		1		1	
Region of residence (tv)	Urban	1		1		1		1		1		1		1		1		1		1	
	Semiurban	1.07		0.98		0.99		1.15		1.22		1.08	***	1.10		0.98		0.96	*	0.91	***
	Rural	0.92		1.07		0.92		0.99		1.22		1.06	***	0.96	*	0.93	***	0.99		1.00	
																					
Months employed prior to 1st birth, female partner	0 months											1.10	***	0.96	*	0.96	*	0.95	*	0.92	**
	1–5 months											0.94	*	0.96		1.09	**	0.96		0.53	**
	6–11 months											0.90	***	0.96		1.05		1.02		0.62	**
	12 months											1		1		1		1		1	
Male partner unemployed (tv)	No	1		1		1		1		1		1		1		1		1		1	
	Yes	1.00		1.01		1.03		0.94		0.99		1.02		1.00		1.02		0.96		1.02	
Income prior to 1st birth, female partner	<10,000											1.56	***	0.94	**	1.00		0.98		0.92	**
	10,000–15,999											1.26	***	0.97		0.99		0.99		0.93	**
	16,000–27,999											1		1		1		1		1	
	≥28,000											0.90	***	1.05	*	1.09	***	1.05	*	1.07	
Age of the youngest child (tv)	0	0.33	***	0.17	*******	0.16	*******	1.50	*******	7.96	*******	1.40	***	0.29	***	0.22	***	0.80	***	1.34	***
	1–2	5.82	***	0.25	*******	0.04	*******	0.23	*******	1.33	******	8.19	***	0.34	***	0.05	***	0.15	***	0.36	***
	3+	1		1		1		1		1		1		1		1		1		1	
Number of children (tv)	1	1		1		1		1		1		1		1		1		1		1	
	2	46.56	***	4.20	*******	4.20	*******	0.35	*******	0.06	*******	4.80	***	1.55	***	1.24	***	0.48	***	0.23	***
	3	1,285.17	***	18.11	*******	16.00	*******	0.21	*******	0.01	*******	24.22	***	2.80	***	2.00	***	0.31	***	0.07	***
	4+	11,008.53	***	104.13	*******	75.45	*******	0.20	*******	0.00	*******	108.88	***	4.69	***	2.40	***	0.18	***	0.02	***
Constant												1.866547	***	0.30	***	0.12	***	0.00	***	0.00	***
Log‐likelihood		−49,391		−63,030		−72,736		−447,645		−19,063		−106,697		−98,088		−113,215		−69,895		−35,534	
LR chi^2^		136,514	***	14,631	***	63,592	***	42,599	***	36,965	***	176,704	***	13,870	***	71,132	***	62,005	***	60,267	***
Observations		264,228		260,800		239,833		171,370		110,423		286,199		286,199		282,704		268,510		239,445	

*Source:* Finnish Register Data, own calculations.

*Note:* Discrete‐time event history and fixed effects discrete‐time event history models for nonrepeated events. All time‐varying variables are lagged.

**p* < .05. ***p* < .01. ****p* < .001.
