# Supplementary material for: Cash‐for‐Care Use and Union Dissolution in Finland
Source: J Marriage Fam. 2020 Nov 17;83(1):209–27. doi: 10.1111/jomf.12738 (PMC7839531; doi:10.1111/jomf.12738)
Supplement: Supplementary file 1 — Appendix S1: Supporting Information [file JOMF-83-209-s001.pdf]

Appendix
Morosow, Jalovaara & Härkönen
Cash-for-Care use and Union Dissolution in Finland
Journal of Marriage and Family
Online only

Figure S1: Union dissolution survival curves of an average couple between 21 and 30, with secondary vocational education, that is married and with a union duration of two years prior to their first child. Discrete-time event history analysis, estimates from Model 3.

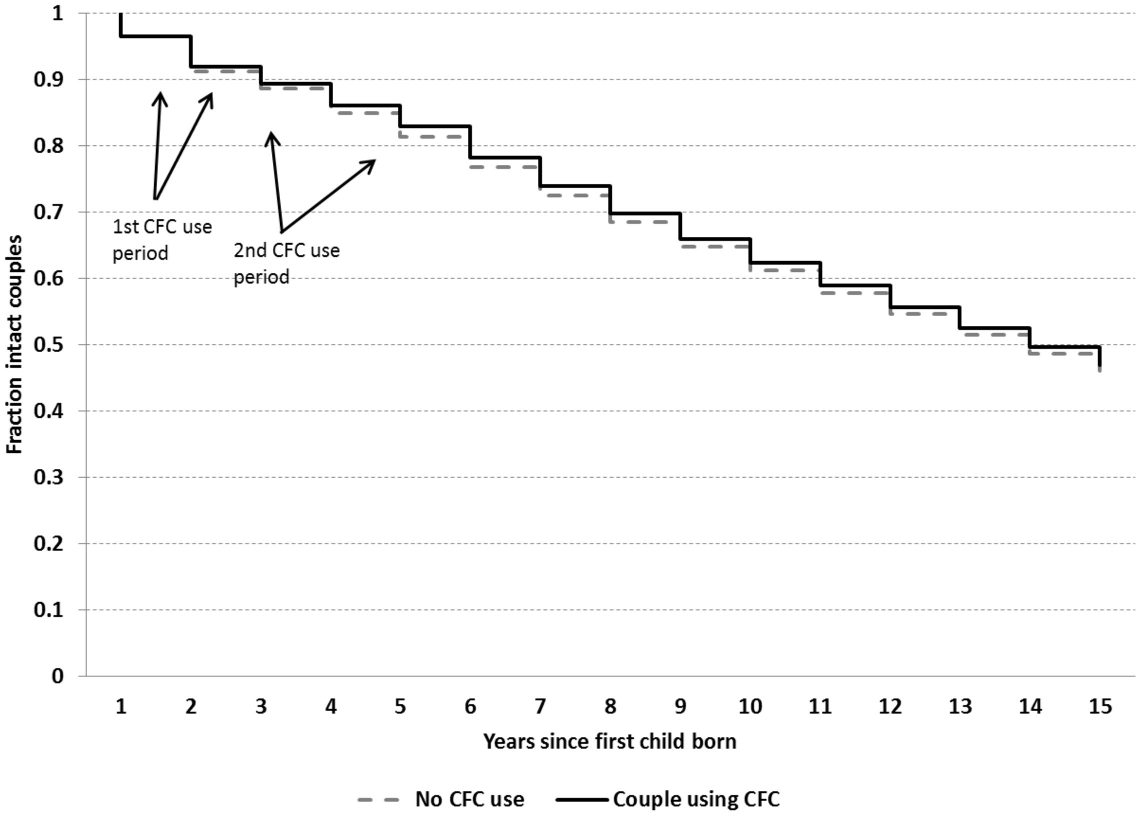

Note (tv: time-varying): controlled for duration, duration squared, period (tv), union duration prior to 1st birth, female partner's age (tv), female partner's education (tv), male partner's education (tv), union type prior to first birth, region of residence (tv), female partner's months employed prior to 1st birth, male partner's unemployment (tv), age of the youngest child (tv), and the number of children (tv).
Source: Finnish register data, own calculations.

Table S1. Results of the discrete-time event history models on separation risk including length of CFC leave, odds ratios.

		Model 1		Model 2		Model 3	
CFC use & time since CFC use (tv)	no CFC	1		1		1	
	currently using CFC	0.81	***	0.75	***	0.87	**
	1 year since CFC	1.06		1.03		1.02	
	2–4 years since CFC	1.13	**	1.07		0.98	
	5–7 years since CFC	1.21	***	1.12	*	1.00	
	8+ years since CFC	1.15	*	1.04		0.90	
Length of CFC use, accumulated (tv)	1	1		1		1	
	2	1.06		1.00		1.05	
	3	1.08	*	0.97		0.99	
	4	0.96		0.86	***	0.94	
	5	0.99		0.81	***	0.89	*
	6+	0.87	***	0.70	***	0.80	**
Duration		0.92	***	0.99		0.99	
Duration squared		1.00	*	1.00		1.00	
Period (tv)	1987-1990	0.97		0.84		0.82	
	1991-1993	0.88	*	0.83	***	0.83	***
	1994-1996	0.79	***	0.77	***	0.78	***
	1997-2000	0.80	***	0.78	***	0.78	***
	2001-2004	0.83	***	0.81	***	0.81	***
	2005-2009	1		1		1	
Union duration prior to 1st birth		0.87	***	0.94	***	0.94	***
Age, female partner (tv)	Under 21			1.85	***	1.84	***
	21-30			1		1	
	31-40			0.78	***	0.74	***
	41-50			0.64	***	0.56	***
	51+			0.47	***	0.39	***
Female partner’s education (tv)	Basic			1.50	***	1.47	***
	Secondary vocational			1		1	
	Secondary academic			1.09	*	1.09	*
	Low tertiary			0.82	***	0.83	***
	High tertiary/University			0.74	***	0.76	***
Male partner's education (tv)	Basic			1.32	***	1.31	***
	Secondary vocational			1		1	
	Secondary academic			0.99		0.99	
	Low tertiary			0.79	***	0.80	***
	High tertiary/University			0.72	***	0.74	***
Union type prior to first birth	Cohabiting			1.28	***	1.24	***
	Married			1		1	
Region of residence (tv)	Urban			1		1	
	Semi-urban			0.76	***	0.77	***
	Rural			0.66	***	0.68	***
Months employed prior to 1st birth, female partner	0 Months			1.14	***	1.14	***
	1-5 Months			1.04		1.03	
	6-11 Months			1.02		1.03	
	12 Months			1		1	

		Model 1		Model 2		Model 3	
Male partner unemployed (tv)	0			1		1	
	1			1.48	***	1.48	***
Income prior to 1st birth, female partner	<10,000			1.21	***	1.20	***
	10,000-15,999			1.10	**	1.09	**
	16,000-27,999			1		1	
	>= 28,000			1.04		1.05	
Age of the youngest child (tv)	0					0.48	***
	1-2					0.76	***
	3+					1	
Number of children (tv)	1					1	
	2					0.81	***
	3					0.73	***
	4+					0.68	***
Constant		0.08	***	0.06	***	0.07	***
Log-Likelihood		-40374.92		-39266.3		-39021.0	
LR chi2				2210.0	***	490.7	***
Observations		312,256		312,256		312,256	

* p<0.05; ** p<0.01; *** p<0.001

Source: Finnish Register Data, own calculations

Note: (tv) indicated time-varying independent variable, all time-varying variables are lagged by one year
